# Patients’ experience of a novel interdisciplinary nurse-led self-management intervention (INSELMA)—a qualitative evaluation

**DOI:** 10.1186/s41927-024-00379-6

**Published:** 2024-03-01

**Authors:** Nadine Schäffer Blum, Bente Appel Esbensen, Mikkel Østergaard, Ann Bremander, Oliver Hendricks, Luise Holberg Lindgren, Lena Andersen, Kim Vilbaek Jensen, Jette Primdahl

**Affiliations:** 1 Copenhagen Center for Arthritis Research (COPECARE), Center for Rheumatology and Spine Diseases, Copenhagen University Hospital Rigshospitalet, Glostrup, Denmark; 2 Department of Clinical Medicine, Faculty of Health and Medical Sciences, University of Copenhagen, Copenhagen, Denmark; 3 Danish Hospital for Rheumatic Diseases, University Hospital of Southern Denmark, Sønderborg, Denmark; 4 Section of Rheumatology, Department of Clinical Sciences Lund, Lund University, Lund, Sweden; 5 Spenshult Research and Development Centre, Halmstad, Sweden; 6 Department of Regional Health Research, University of Southern Denmark, Odense, Denmark; 7 University Hospital of Southern Denmark, Aabenraa, Denmark; 8 Patient research partner, Nyborg, Denmark; 9 Patient research partner, Helsingør, Denmark

**Keywords:** Qualitative research, Health literacy, Self-efficacy, Complex intervention, Rehabilitation, Person-centred, Goal setting, PSFS

## Abstract

**Background:**

Despite continuous improvements in anti-rheumatic pharmacological treatment, people with chronic inflammatory arthritis still report substantial disease impact. Based on the framework for complex interventions, we thus developed INSELMA, a novel nurse-coordinated multidisciplinary self-management intervention for patients with rheumatoid arthritis, psoriatic arthritis or axial spondyloarthritis. Based on individual biopsychosocial assessments, a rheumatology nurse facilitated goal setting and coordinated interdisciplinary support. The aim of this study was to explore the patients’ experience of participating in the six-months INSELMA intervention.

**Methods:**

Individual semi-structured interviews were conducted with 15 of the participants after their final follow-up. Thematic analysis was applied.

**Results:**

The analysis derived four overall themes. (1) *A new opportunity at the right time*. The participants’ disease impacted all areas of daily life. Participation in INSELMA was experienced as an opportunity to improve symptoms and together reduce long-held challenges they had fought alone, until now. (2) *The importance of person-centred goals*. The participants found it meaningful to work with their individual goals, which encompassed physical, psychological, and social factors. Having time between consultations to work with goals at home was important. (3) *Empathy, partnership and a little nudging from health professionals are essential*. The empathic nurses’ continuous support and coaching helped participants become aware of their own resources. The participants highlighted having access to support from a physiotherapist and occupational therapist with rheumatology experience as important. (4) *I got more than I could have hoped for*. Most of the participants experienced decreased symptom load and improvement in physical strength, mobility, sleep, and mood as well as increased energy, knowledge, and self-management ability. The participants expressed new hope for the future with an improved ability to manage their symptoms and work towards new goals.

**Conclusion:**

The participants found the INSELMA intervention meaningful and feasible. They experienced decreased disease impact and increased activity levels, facilitated by empathy and self-management support from health professionals.

**Supplementary Information:**

The online version contains supplementary material available at 10.1186/s41927-024-00379-6.

## Background

Autoimmune chronic inflammatory arthritis (IA) includes rheumatoid arthritis (RA), psoriatic arthritis (PsA), and axial spondyloarthritis (axSpA) [1]. These diseases are characterised by recurrent periods of inflammation, swelling, and tenderness of peripheral and/or axial joints. If left untreated, IA can lead to joint damage, deformities, and loss of function [2–4]. IA is associated with a higher risk of comorbidities, such as cardiovascular disease, diabetes, osteoporosis, infections, certain types of cancer, depression, anxiety, and overall increased mortality [5–7].

Despite a ‘treat-to-target’ strategy with the aim of achieving remission or low disease activity [8], many patients with IA still experience a significant impact of their arthritis [9–11]. Up to 40% of patients do not reach the criteria for clinical remission and many in remission or with low disease activity state still report a substantial impact [12–14]. Fluctuating symptoms such as morning stiffness, physical limitations, pain, fatigue, sleep disturbances, anxiety and depressed mood, affect patients’ daily lives, including their ability to work, further social participation, and quality of life (QoL) [15–17].

The European Alliance of Associations for Rheumatology (EULAR) recommends a biopsychosocial approach that involves both the rheumatology nurse and the multidisciplinary team in the care of the patients [18]. In addition, EULAR recommends self-management support as a core strategy to empower patients to live with IA and reduce the impact of their disease [19].

Self-management has been defined by Barlow as *“the individual’s ability to manage the symptoms, treatment, physical and psychosocial consequences, and lifestyle changes inherent in living with a chronic condition.”* [20]. Self-management skills involve concordance, goal setting, and problem-solving, and impact medication adherence, healthy lifestyle choices and the patient’s QoL [21]. Self-management skills are mediated by health literacy and self-efficacy [22]. Health literacy covers the cognitive and social skills required to access, comprehend, evaluate, and apply health-related information [23]. Self-efficacy refers to individuals’ perceptions of their own ability to manage or solve specific tasks or problems [24, 25].

The evidence from outpatient self-management interventions for people with IA varies and is inconsistent and individually tailored interventions are necessary [26] Therefore we developed INSELMA [27], a novel interdisciplinary nurse-led self-management intervention based on the British Medical Research Council (MRC) framework for developing and evaluating complex interventions [28]. The self-management intervention targets patients with IA and substantial disease impact. Furthermore, the intervention is based on three theoretical and conceptual approaches: acceptance and commitment therapy (ACT), self-efficacy, and health literacy [23, 25, 29]. The comprehensive development of the INSELMA intervention is described in detail elsewhere [27]. A six-month feasibility study was carried out from February 2022 to January 2023. A total of 18 participants were initially included in the feasibility study. Subsequently, one participant withdrew following the initial consultation due to time constraints. The remaining 17 participants completed the intervention. To evaluate the acceptability and feasibility of the intervention, it is important to include the participants’ perspectives. Thus, the aim of this study was to explore the patients’ experience of participating in the six-months INSELMA intervention. The experience of the health professionals (HPs) will be reported elsewhere.

## Methods

### Study design

This study was planned as a qualitative study based on individual semi-structured interviews with patients who had participated in the INSELMA feasibility test.

### Setting

Two rheumatology outpatient clinics in Denmark were involved in the development and feasibility testing of INSELMA, namely, the Danish Hospital for Rheumatic Diseases, Sønderborg and the Centre of Rheumatology and Spine Diseases at Copenhagen University Hospital, Rigshospitalet-Glostrup. The research group comprised researchers with diverse professional backgrounds (two rheumatologists, a physiotherapist, three nurses) and two patient research partners.

The research group defined the following research questions to be able to evaluate feasibility of the INSELMA intervention and how to improve it further:

1. What were the patients’ expectations and motivation for participation in the intervention?
2. How did the participants experience the outline of the intervention regarding the initial assessment, goal setting, communication, collaboration with the HPs and coherence between primary and secondary care?
3. What were the experienced impact, and mode of impact, from participating in INSELMA?

### The interdisciplinary nurse-coordinated SELf-MAnagement (INSELMA) intervention

INSELMA aimed to support the self-management ability of patients living with a substantial disease impact [27]. Patients over 18 years of age, who had lived with RA, PsA or axSpA for at least 24 months, were recruited through the national Danish Rheumatology Database DANBIO [30]. Patients were eligible if they had either answered ‘no’ on the Patient Acceptable Symptom Scale (PASS) [31], and/or had graded their symptoms at 40 or above on a visual analogue scale (VAS 0-100) on at least two measures in fatigue, pain or patient global assessment (PGA) [32]. Exclusion criteria were cognitive problems, potential changes in pharmacological treatment, participation in other rehabilitation interventions and research studies, or ongoing applications for pension.

Each participant was assigned to an experienced coordinating rheumatology nurse (CRN) who performed an initial biopsychosocial assessment and followed a goal setting process using communication tools from a comprehensive intervention manual and i.e. elements from training in an Acceptance and Commitment Therapy (ACT) [33], self-efficacy and Health Literacy. The goal-setting process was based on shared decision-making [34]. The participants defined individual goals and up to five activities, using the Patient Specific Functional Scale (PSFS) [35, 36], that they would like to improve, and which were both important and challenging to them. Each participant was assigned 2.5 h of individual support from the CRN, either by phone, face-to-face, or online. The CRN would coordinate relevant support from interdisciplinary partners, e.g., physiotherapist, occupational therapist, or a social worker based either at the hospital or in primary health care. At each face-to-face meeting with the CRN, the goals, and PSFS activities were evaluated. The final consultation would include a recap and a discussion of the participant’s future need for support.

### Participants

The participants were recruited consecutively for an interview, two weeks after their final consultation in the feasibility study. The interviewer contacted the participants by telephone to agree on a time and form for the interview, either by phone or face-to-face at one of the hospitals depending on the participants’ preferences. Prior to the interviews, the participants received additional written information by email about the aim and setting for the interview, along with the interviewer’s name and affiliation.

### Data collection

The research group developed a semi-structured interview guide (Supplementary file 1) based on the research questions and the intervention manual. The interview guide consisted of open-ended questions to uncover the participants’ experiences of the intervention in relation to the aim of the study. The interviewer used probing questions to explore the participants’ experience. Table 1 shows examples of questions that were used during the interviews. The interviewer remained open to further explore experiences that emerged as important for the individual participant.

**Table 1:** The overall topics in the interview guide

Topics	Examples of questions
Before intervention	What were your thoughts when entering the intervention?
The initial consultation	How did you experience the first consultation with the coordinating rheumatology nurse?
During intervention	What has changed for you during the intervention? What made a difference for you?
After intervention	What significance did the intervention have for you?
Your future	How do you feel able to manage your IA and everyday life in the future compared to the time before participating in INSELMA?
Other	What do you think could have been done differently?

The interviews were conducted by the first author (NB), who is a female research assistant, registered nurse with experience as a clinical nurse, a master’s degree in Health Science, and is experienced in conducting qualitative interviews. The interviewer was not involved in the development or delivery of the intervention. Each interview was recorded and transcribed verbatim by the interviewer.

### Patient research partners

Two patient research partners (PRPs) participated in accordance with the EULAR recommendations for the inclusion of patients in scientific projects [37]. The collaboration with the PRPs is reported in accordance with the Guidance for Reporting Involvement of Patients and the Public [38]. The PRPs (LA, KVJ) were a man and a woman with RA, one from each hospital, and both experienced substantial impact of their arthritis. They were part of the overall project group and participated in all phases of the overall INSELMA study; the development of the intervention study [27], the intervention, the manual, and the interview guide. They contributed to the interpretation of the raw data from the interviews and commented on and accepted the final manuscript. In addition, the study and its preliminary results were discussed and received very positively by the research user council at the Danish Hospital for Rheumatic Diseases, which comprises eight patient representatives with RA, PsA or axSpA.

### Data analysis

Reflexive thematic analysis, as described by Braun and Clarke was applied [39, 40]. Table 2 shows an overview of the steps in the analysis. The analysis was a combination of an inductive and a deductive approach, which implies that, while the developed themes were connected to the theoretical background, they were also grounded in the raw data.

**Table 2:** Steps in reflexive thematic analysis [40]

Analytical phases		Description of analysis process with examples from the study
1	Dataset familiarisation	The research group read, and the first author reread all interviews to become familiar with the overall content
2	Generation of initial codes	The first author identified concepts potentially interesting for the research question. e.g.: *- I won’t let IA win over me* *- Goal setting was complicated* *- I felt heard and understood*
3	Organisation of codes	Codes were collected into initial themes, i.e.: *- Reasons for participating; expectations, hopes, thoughts.* *- Experience of process, goal setting and shared decision making.* *- Experience of change and expectations for one’s own future* *- Suggestion for future projects* *- Context. Daily life living with IA*
4	Review and revise themes	Themes were revised to ensure that they highlighted patterns in the data. This included division and merging of themes
5	Define naming of themes	Ensured that the name of the theme told a concise story of the theme e.g., *‘Someone who walks with me, holds my hand or pushes me in the right direction’*, which progressed to the theme: ‘*Empathy, partnership and a little nudging from HPs are essential*’
6	Write up the results	Results were written in narrative text supported by quotations

We used the NVivo qualitative data analysis software (QSR NVivo v20.6.1.1137) to support the analysis. The initial coding and primary analyses were conducted by the first author in close dialogue with two of the researchers (JP, BAE). The analysis and results were discussed with the entire research group.

The study is reported in accordance with the Consolidated Criteria for Reporting Qualitative Studies (COREQ) [41].

## Results

Seventeen participants completed the intervention. Two female participants did not respond to repeated invitations for an interview. Thus, fifteen semi-structured interviews were conducted, with nine women and six men, from September 2022 to January 2023. Two male participants chose to be interviewed face-to-face, while the rest preferred to participate in an interview by phone. The interviews lasted between 18 and 82 min, with a median of 26 min. The characteristics of the interviewed participants are described in Table 3 below.

**Table 3:** Baseline characteristics of the interviewed participants

Socio-demographics	*N* = 15	
Age, median (IQR)*	56 (49;71)	
Sex, female, n (%)	9 (60)	
Cohabiting, n (%)	8 (53)	
Current connection to labour market, n (%)	5 (33)	
Unemployed, n (%)	1 (7)	
Early retirement, n (%)	4 (27)	
Pension, n (%)	5 (33)	
**Disease related information**		
Rheumatoid arthritis, n (%)	6 (40)	
Psoriatic arthritis, n (%)	5 (33)	
Axial spondyloarthritis, n (%)	4 (27)	
Years living with IA, median (IQR)	13 (6;16)	
Self-reported comorbidity, n (%)	12 (80)	
**Selected PROMs**		
Disease impact, VAS** 0-100, median (IQR)	71 (57;79)	
Pain, VAS 0-100, median (IQR)	68 (54;75)	
Fatigue, VAS 0-100, median (IQR)	75 (58;92)	

*IQR = Interquartile range; n = number; IA = inflammatory Arthritis; PROMs = Patient Reported Outcome Measures; **VAS = Visual Analogue Scale anchored by ‘No impact/pain/fatigue’ to ‘Unbearable impact/pain/fatigue’

The analysis derived four themes: (1) A new opportunity at the right time; (2) The importance of person-centred goals; (3) Empathy, partnership and a little nudging from HPs are essential; (4) I got more than I could have hoped for. Each theme is described in detail below and illustrated by selected quotations.

### A new opportunity at the right time

The participants shared their life circumstances, including the onset and progression of their arthritis over the years. They found it difficult to recall the initial consultation with the CRN, but described how their IA affected all aspects of their lives before the INSELMA intervention, with pain, fatigue and physical restrictions dominating their lives. Several expressed that their mood had been negatively affected and described anxiety, depressive, or suicidal thoughts due to life traumas or severe pain, which made them feel that their life had become meaningless. The male participants talked about isolation and feeling very lonely due to invisible symptoms and lack of understanding from those in their familial network, while the female participants experienced social support. However, some of the female participants found it difficult to set boundaries to take care of themselves, because of difficult life events or challenging family affairs. The participants who were still at the labour market, described how their arthritis affected their work life, leading to loss of identity. Several had taken early retirement due to their arthritis, had been fired, reduced working hours or had changed jobs because of their arthritis. The participants revealed different strategies to manage their life with IA. Some participants took an active fighter approach: *“I won’t let it win over me’”*, *“I do what I know is good for me”* or *“I want to take care of myself without help”*, while others took a more passive or accepting approach, saying *“After so many years, I have adjusted my life and learned to live with it.”*

Their long-held challenges motivated them to participate in the INSELMA intervention, even though some worried their situation might be too complex. For a few, the motivation to participate represented a chance to contribute to research to improve future IA treatment for others. Many expressed a feeling that they felt they had been fighting their problems alone. They had yearned for change for a long time but had felt unable to do something about their situation themselves. The participants felt the offer to participate in INSELMA came at just the right time in their lives when they felt burdened physically, socially, and emotionally.

*I started to cry when I got the offer. (…) When you guys called me, I was actually worn out in general. My thoughts revolved that FINALLY someone will listen to me. Maybe this time I won’t hit the wall*. (Female, age 40–45 years, ID5)

The intervention provided a way to target challenges that they did not have energy or abilities to manage themselves before participation in INSELMA.

*My motivation was that I was at a time in my life, when I thought everything was a burden, as I gave IA the blame for everything. (…) Something else had to happen to get me out of the deadlock I was in*. (Male, age 50–55 years, ID6)

For some, participation in the INSELMA intervention was a cherished opportunity to receive professional support from the HPs to manage specific problems or improve their general wellbeing.

*My expectation was not to become Superman, but just to get started and to implement working out twice a week*. (Male, age 45–50 years, ID7)

### The importance of person-centred goals

The outline of the intervention was experienced as highly acceptable, meaningful, and relevant. The participants experienced the goal setting process, with defining and working with activities as very person-centred because they focused on what was most important to them. The activities and goals defined during the initial consultation varied according to the impact on their daily lives. Many of the participants had a clear vision of what they wanted to work with when they started the intervention.

*I already knew beforehand what I wanted. I had given it a lot of thought. (…) Primarily being more pain free. (…) It was my focus. (…) How can I make my life easier with assistive devices? To wash my hair when in pain, it seems ridiculous. There were some basic things I could not do, when I first met with them, that I needed help with*. (Female, age 40–45 years, ID5)

A few participants expressed that it had been difficult to define the activities that were specific enough to enable evaluation over time. These participants had chosen activities that were not quite realistic to achieve, and one expressed having too high expectations of the possible benefit from participating in the intervention.

The activities and goals defined during the initial consultation varied according to the impact on their daily lives. All participants had defined activities related to the physical impact of their arthritis, such as an intention to increase physical activity level or improve activities of daily living related to a painful limb. In addition, many wanted to work with self-management of fatigue or pain. Some had defined goals related to improvement of their social life, such as to see more people or practice setting boundaries and communicating the boundaries in their social network.

Most participants had been referred to a physiotherapist and/or an occupational therapist with experience in rheumatology at the hospital as part of the intervention and some had contact with a podiatrist or a psychologist in primary care. The opportunity to be referred to a rheumatology physiotherapist or occupational therapist as part of the INSELMA intervention was highlighted as being of great importance for achieving improvements. The therapists provided the participants with individual exercise plans, followed up on agreements, expressed empathy and stimulated the participants’ motivation for change. Furthermore, the therapists referred participants to further interventions or contacts in primary health care, and the occupational therapists supported the participants who needed to apply for aid in the municipality. The participants experienced that the interdisciplinary team worked together, while focusing on different aspects, which improved the overall outcomes.

*I think it was great to be able to have the combination of physiotherapy and occupational therapy. (...) They also discussed with each other. (...) ‘How can we get there?’ (…) It was a group of competent people who could help me achieve what I wanted. I also told the nurse, when it was over, that it was a real shame that it didn’t continue*. (Female, age 45–50 years, ID10)

The participants found the duration of the intervention appropriate to achieve a positive outcome and reach their goals. Some expressed a desire to continue working towards new goals, while others had concerns about difficulties staying on the right track without continuous support from the HPs in the future. While one participant suggested shorter or more intense courses targeting specific issues, most of the participants wished to continue with the consultations, either in fixed intervals or for shorter periods of time when needed. The participants were content with the frequency of consultations. Some expressed that it was important to have time between the consultations to work on their defined activities at home, to experience progress.

*We saw each other (...) almost once a month. (...) It was appropriate, because you had time to improve and write down potential questions you had during that time. (…) Then, we had a measurement parameter on how much I was able to do and not do.* (Female, age 40–45 years, ID5)

### Empathy, partnership and a little nudging means everything

The participants' experience of an empathic and trusting professional relationship with the HPs was emphasised as being of the utmost importance and an essential part of what made a difference to them in the intervention. All the participants expressed that the assigned nurses were able to create a safe space in which they felt comfortable talking about vulnerable issues. This encompassed their arthritis and mental health, as well as their overall life situation, including family issues that could affect their ability to take care of themselves. Some participants described the CRN as a companion that would take them by the hand or ear, walk with them and help keeping them on the right track. They highlighted that the interpersonal relation to the CRN influenced the how much they gained from the intervention.

*And it helped me. Especially these conversations with the nurse. As a light to look forward to. I think* [the nurse] *was fantastic to talk to and* [the physiotherapist] *as well. Very empathic. I was relieved, happy and in a good mood*.

(Male, age 70–75 years, ID9)

The participants greatly valued the ongoing support from the same CRN, who demonstrated dedication, empathy, expertise, knowledge, and consistency in addressing their specific needs. They perceived that they had a collaborative partnership with the HPs in general, where they felt seen, heard, understood, acknowledged, and validated with respect to their challenges perhaps for the first time since being diagnosed. This instilled hope for the future.

*Well, this* [INSELMA] *has meant everything. Because my own rheumatologist said: ‘Well, we cannot do more. We cannot increase the pills.’ (…) Now, here was someone saying: ‘Can we do something? Can we improve it* [disease impact]*?* (Female, age 70–75 years, ID11)

The participants considered that the initial consultation with the CRN should be face-to-face to establish a confidential relationship. Those with multiple symptoms, long travel time, or an active work life, appreciated the opportunity for follow-up by telephone. However, participants who expressed loneliness, anxiety, or psychosocial problems preferred face-to-face meetings, as they valued eye-contact and the ability to read the nurse’s body language. In addition, the participants expressed that assessments and consultations with the therapists needed to be face-to-face.

Some participants reflected on their own responsibility. They expressed that their expectations towards the content, goals and possible outcome had to be realistic, achievable, and aligned with those of the HPs. They considered that it was necessary to feel truly motivated and ready to engage and do the work necessary to experience progress and benefit from the intervention. Some reflected upon the possibility of group sessions as part of the intervention. While some were open to group sessions as a possibility to share experiences, several emphasised that they preferred not to participate in groups, because their problems were too sensitive and personal to be discussed in plenum.

The consultations with the CRN enabled the participants to reflect on their expectations, values, life situation, and habits. Some mentioned that the conversations helped them acknowledge their own strengths and resources, which further motivated them and led them to change their mindset regarding their life situation.

The participants especially appreciated the CRNs’ communication skills. A few noticed that the CRN had not fully incorporated the new conversation techniques (the ACT principles) in their natural vocabulary.

*The nurse asked in a way that made me dig a little deeper and find more that I had pushed aside and just given up on. (...) We actually worked with that. Here was someone cheering on you saying: ‘Now you do something about it. Now we will try to do something about it.*’ (Female, age 45–50 years, ID10)

### I got more than I could have hoped for

In general, the participants were delighted to have participated in the INSELMA intervention. Several expressed gratitude and became emotional when talking about the positive impact the intervention had on their lives on both their physical function and mental wellbeing.

*It* [INSELMA] *has improved my quality of life by 99%! (.) When you feel that you can only sit on the couch and watch TV, walk with crutches to the kitchen (...) or go shopping with a walker. (…) Suddenly, now I can walk, bike and everything. (…) I sleep better at night. (…) When my grandchildren say: ‘Wow Dad, have you seen how fast grandma is walking now?’ That is proof that others notice that it has helped.* (Female, age 70–75 years, ID11)

The majority of the participants reported a positive development during the intervention period, where they succeeded to implement new habits to achieve their goals and defined activities. Many participants reported less impact of the disease such as reduced morning stiffness, improved mobility, lower pain level, increased physical activity, improved mood and quality of sleep and less fatigue. Some participants told that they had reduced the use of painkillers.

*On a pain scale from 1 to 10 I was sometimes around 7–8. Now I am down between 1 and 3. I don’t think I will get out of it [analgetic], but it is possible now to live with it.* (Female, age 60–65, ID16)

Two participants expressed they did not gain anything from the intervention, that resembled their regular consultations in clinical practice with pleasant conversations with empathetic HPs. However, they both reported changes in their lifestyle habits regarding physical activity.

*I did not gain anything, but then again, maybe I had wrong expectations. (…) I got this out of it that I pushed myself to start working out. By myself and in gym classes. (…) The* [INSELMA] *invitation got me to start again.* (Male, age 70–75, ID3)

Several of the participants talked about a positive change in their mindset. For some, this included improved communication with their family and to set boundaries to take care of their own needs, which relieved them from feeling pressured by social expectations. Some expressed having worked with their self-image to adjust their own expectations of their abilities, which reduced their feelings of guilt of not doing enough. As a result, they experienced improved coping and mental well-being.

*We started making some* [goals] *about my fatigue and ability to plan my day. (…) It quickly became clear that it was not the main problem. (…) What frustrated me was that I expected to be able to do more. (…) I was that kind of person. I had not fully accepted that I had a chronic disease. (…) There are always some superhumans in the world (...) and compared to them, you can’t do as much. (…) This is where I think the project made a clear difference. I have found a realistic approach to what I can do, but also realised that I did more than I thought.* (Male, age 50–55 years, ID6)

All the participants had lived with IA for many years and appreciated the opportunity to refresh or gain new knowledge about their arthritis and how to improve life with the chronic condition. Some had already been very active in seeking information online or on social media about self-management of their symptoms before participating in the intervention, but they found it difficult to find useful and trustworthy information. The INSELMA intervention made them aware of opportunities to join patient groups or avail of other support opportunities in primary care.

*This course refreshed what I learned many years ago, but never managed to do something about. (…) Now I use the arthritis association. I have become aware that they have many events.* (Female, age 60–65 years, ID8)

Some participants reflected on their overall experience of the healthcare system. As many lived with comorbidities and had struggled to navigate the system, they felt that the INSELMA intervention provided a holistic assessment where all aspects of their life situation were taken into consideration and put into an overall context.

*It was like an overall view on me as a person. Because I think that many of the problems, I have had in the last (...) 9 years could have been avoided if one* [the system] *had listened to me to begin with and perhaps taken a holistic view of me as a person and had not only looked at the individual joint*.

(Female, age 40–45 years, ID5)

The participants appreciated the assessments and exercises provided by the physiotherapists, which, in combination with emotional support from the nurses, helped them become more aware of reasons to be and how to be physically active or understand that physical activity encompasses more than planned physical exercise. Almost all participants told that they had increased their physical activity level after participating in INSELMA. They highlighted the benefits of physical activity for not only symptom management but also their general well-being. Some participants shared that they had adopted new habits during the intervention. These included joining the local gym, fixed agreements with family members about joint exercise or taking initiatives in local IA associations. They felt more motivated and capable of taking action in the future.

*I am surprised how deeply we got into what really bothered me in my daily life. (…) What surprised me the most is that I came out of this thinking I have gained so much. I did not expect that. I thought ‘well I will give it a shot. It might help’. However, it has definitely had an impact on me, going home and making appointments with the family. It is giving all of us something. We are together about things now.*

(Female, age 45–50 years, ID10)

## Discussion

The aim of this study was to explore the experience of participating in the INSELMA intervention. Overall, the participants expressed a very positive attitude towards all parts of the self-management intervention. They were grateful to have been given the opportunity to participate and they found the intervention to be relevant, meaningful, and beneficial. The findings demonstrate a high degree of acceptability among a group of patients who experienced a considerable impact of their arthritis. It suggests that the INSELMA intervention is a feasible way to target the participants’ self-management ability and reduce the impact of their IA.

The program theory behind INSELMA revolves around a basic logic model defining both the planned intervention and the intended results [27]. I.e., we assumed that participants would experience a person-centred approach, feel acknowledged and have their needs met. Moreover, we expected that the participants would experience continuity in care and that the self-management support would facilitate behavioural change, improve symptom management, and reduce the impact of fatigue, pain, and sleep problems. We also expected that they might experience increased mental wellbeing, health-related QoL and physical activity, as well as lower absenteeism from their paid work. According to the overall qualitative evaluation, the intervention fulfils its purpose, and the findings support the logic model.

Our qualitative findings indicate that we managed to include a group of patients who lived with significant impact on their everyday life due to IA despite the optimal pharmacological treatment. The participants considered that the intervention was offered at a time in their life, when they needed it the most. The participants had lived with their unmet needs for such a long time, that they were unable to target and alleviate themselves and would have appreciated help years before. The participants’ positive experiences from participation in the INSELMA intervention suggest that a comprehensive and interprofessional biopsychosocial approach combining physical therapy, occupational therapy, nursing, and psychologically informed support can lead to improved overall well-being for people living with substantial impact of IA.

It is important to acknowledge that the feasibility test was not powered nor designed to measure effect, but the perceptions of the participants show a potential benefit on the disease related impact. The participants reported a reduction in symptoms, such as pain, fatigue, and emotional distress. They experienced that they got new knowledge, tools, and support to utilise resources and implement new habits to manage their life with IA, especially regarding physical activity and pain management. This highlights the empowering nature of the INSELMA intervention. This resonates with findings from other trials that have found that self-management interventions and nurse-led patient education led to significant improvements in symptoms such as fatigue, pain, sleep problems, anxiety and depression along with coping skills, self-efficacy, illness perception and QoL, with effects lasting over time [22, 42–45]. Regarding absenteeism, only a few of the participants were in paid work, but they prioritised work and tried to not be absent despite the challenges caused by their IA. This confirms the findings from other qualitative studies [46, 47].

The literature shows that patients with IA ask for care that is based on shared decision-making and continuity, and that they have a need for individual and emotional support from HPs [26, 49]. These needs were met in the INSELMA intervention. The participants found it meaningful, relevant, and valuable to work with activities that were person-centred in a process facilitated by the CRNs. The open questions and the discussion of values in accordance with an ACT approach was experienced very positively although a few experienced that some of the CRNs had not fully incorporated these principles yet.

The participants expressed improvement in social functioning, including better communication with HPs, increased social support, and an enhanced ability to engage in social activities, which highlights a potential broader social benefits of self-management programmes such as INSELMA. The improved ability to communicate with HPs may also indicate improved health literacy skills in this area.

The participants emphasised the significance of empathic and acknowledging approach. Other studies have also found that to establish person-centeredness, patients have to feel able to discuss their own ideas about self-care actions with a HP who is willing to listen and has enough time [48–50].

The HPs biopsychosocial approach and empathic attitude in INSELMA made the participants reflect upon their lives, experience the approach as holistic, and a trusting partnership was established. This supported the participants’ confidence and ability to self-manage their symptoms. These findings resonate with other qualitative studies concerning patients with IA or other chronic disease that found that a partnership with HPs may improve the patient’s ability to gain knowledge and manage living with chronic diseases [51, 52]. The patient-nurse relationship builds up over time and the continuity reduces the need for the patients to re-explain their needs, which increases their trust and confidence in the HPs [52]. In our study, the trusting partnership with the CRN made the participants able to work towards their goals together with the interdisciplinary team as well as to make use of relevant opportunities in primary care.

The involvement of rheumatology therapists as part of the interdisciplinary team contributed to positive experiences. This supports that an integrated approach with interdisciplinary involvement to improve overall well-being and self-management in individuals with IA. The participants were satisfied with the opportunity for individually targeted support which increased their engagement. Some suggested that part of the face-to-face and telephone consultations may be replaced by online consultations. The participants who were in paid work appreciated the chance to have follow-up over the phone after the initial face-to-face meeting, whereas participants with substantial emotional impact or a limited social network preferred face-to-face meetings, to enable emotional support. The participants also considered that meetings with the PTs and OTs needed to be face-to-face. This highlights the need for flexible solutions in clinical practice as other studies have found that, e.g., online self-management support can be useful, but not for all [53, 54].

The duration of the intervention was well received by the participants. The participants acknowledged the need for ongoing support and reinforcement to sustain the achieved improvements and prevent relapse, suggesting that long term support and follow-up may be beneficial. Future studies should explore the long-term effects and cost effectiveness of such an outpatient intervention to assess the sustainability of the achieved outcomes and identify potential factors contributing to relapse or maintenance of improvements.

The participants’ experiences were positive across age, diagnoses, disease duration and disease impact. Apart from suggesting that the duration may be individually adapted, the participants had very few ideas for improvement of the intervention. They did have a point about recruitment of patients for future testing of the INSELMA intervention in a larger study. The participants pointed out that it is important to strive for a high information level adapted to the participants’ health literacy levels and to align expectations, so the participants know what to expect and what is required of them. The participants considered that it requires a degree of inner motivation, empowerment, and self-efficacy and a willingness to take ownership and change behaviour. Some of the participants did not know what to expect of the intervention and found it difficult to define realistic goals and activities. These participants did not experience a gain, compared to the participants who started with a clear goal in mind. Thus, the CRNs may need further training in goal setting to facilitate a fruitful process for all participants.

The results from the collected PRO data and the HP experiences from delivering the INSELMA intervention in the feasibility study will be reported elsewhere. It will be interesting to explore whether the quantitative and qualitative results support each other and how they can be used to adjust and improve the INSELMA intervention and training of the HPs.

### Strengths and limitations

There are several strengths to this study. The interviewer (NB) performed all interviews, which ensured homogeneity in the data collection. NB was not involved in the development or delivery of the intervention or in clinical practice, which limited the influence of preconceptions and helped ensure the external validity of the study. NB also performed the initial analysis. Knowing the tone of voice and the atmosphere during the interviews contributed to a deeper understanding of the text, which contributed to internal validity.

The research team encompassed a representation of physiotherapists, nurses and rheumatologists and researchers with extensive experience and knowledge regarding arthritis, self-management, and qualitative research methods. Furthermore, the two PRPs were involved in all phases of the study. They read anonymised transcripts and contributed with input based on their own experiences throughout the study. This validated our findings and various formulations were adapted because of their input.

The interview guide was developed with valuable input from the PRPs and the rest of the project group, which ensured that relevant and important perspectives were included.

The semi-structured interview approach gave the participants the opportunity to reflect on their life with IA before, during and after the intervention and this approach is considered suitable to an assessment of acceptability of the intervention [55]. It could be considered a limitation that participants had difficulties remembering the initial consultation with the CRN six months prior to the interview. The experience of the six months may be influenced by the perceived outcome they experienced. If our aim was to explore their preliminary expectations and their initial impression, we could have conducted an initial interview in the beginning of the intervention or implement a question asked during the initial consultation targeting this specific topic. However, the participants vividly remembered the difficulties that motivated them to participate in the intervention study.

The participants were invited to be interviewed by phone or face-to-face. The lack of body language and eye contact during phone interviews may be considered a limitation as it is important for the relation. However, in the telephone interviews our participants richly shared experiences about vulnerable topics with audible emotion. The opportunity for a telephone interview offered flexibility for the participants and less use of time, which may have increased the number of participants who agreed to participate in an interview. Also, the two methods are considered to correspond regarding uncovering nuances and sensitive subjects [56]. To ensure a variety of perspectives, we aimed to interview all participants in the feasibility study. In total, 15 of the 17 participants who completed the feasibility study agreed to participate in the interview, which we consider to be an acceptable number to ensure validity and information power [57]. We do not know how the two patients who had completed the intervention, but did not respond to an invitation for an interview, experienced their participation.

## Conclusion

This qualitative evaluation study showed that the participants experienced the INSELMA intervention as relevant, beneficial, and feasible, with a high degree of acceptability. The participants experienced a person-centred approach and valued working with their individual goals and activities, and the majority reported that they experienced a positive impact both physically, socially, and/or mentally. The positive experiences were facilitated by the empathic and trusting continuous support and nudging to change behaviour from the CRNs and access to therapists who had rheumatology experience. The findings did not reveal a need for changes of the intervention, but a need for additional training of the HPs. This study supports further testing of the intervention in a larger population, to further document the effect and cost-effectiveness of INSELMA.

### Electronic supplementary material

Below is the link to the electronic supplementary material.

Supplementary Material 1

## Data Availability

The data that support the findings of this study are available upon reasonable request from the corresponding author. The data are not publicly available because of restrictions regarding information that could compromise the privacy of research participants. Data are available until five years after publication, at which time they should be destroyed, in accordance with the approval from the Danish Data Protection Agency.

## Abbreviations

ACT: Acceptance and Commitment Therapy
AxSpA: Axial spondyloarthritis
CRN: Coordinating Rheumatology Nurse
DANBIO: The Danish Rheumatology Database; a national research registry
EULAR: European Alliance of Associations for Rheumatology
HP: Health professional
IA: Chronic inflammatory arthritis
INSELMA: Interdisciplinary Nurse-coordinated SELf-MAnagement intervention
PASS: Patient Acceptable Symptom Scale
PGA: Patient Global Assessment
PROM: Patient Reported Outcome Measure
PRP: Patient Research Partner
PsA: Psoriatic arthritis
PSFS: Patient Specific Functional Scale
RA: Rheumatoid arthritis
VAS: Visual Analogue Scale
QoL: Quality of life

## References

[1] Ledingham J, Snowden N, Ide Z (2017). Diagnosis and early management of inflammatory arthritis. BMJ.

[2] Smolen JS, Aletaha D, Barton A, Burmester GR, Emery P, Firestein GS (2018). Rheumatoid arthritis. Nat Rev Dis Primer.

[3] Sieper J, Poddubnyy D (2017). Axial spondyloarthritis. Lancet Lond Engl.

[4] Ritchlin CT, Colbert RA, Gladman DD (2017). Psoriatic arthritis. N Engl J Med.

[5] Scriffignano S, Perrotta FM, De Socio A, Lubrano E (2019). Role of comorbidities in spondyloarthritis including psoriatic arthritis. Clin Rheumatol.

[6] Figus FA, Piga M, Azzolin I, McConnell R, Iagnocco A (2021). Rheumatoid arthritis: extra-articular manifestations and comorbidities. Autoimmun Rev.

[7] Vallerand IA, Patten SB, Barnabe C (2019). Depression and the risk of rheumatoid arthritis. Curr Opin Rheumatol.

[8] Smolen JS (2016). Treat-to-target as an approach in inflammatory arthritis. Curr Opin Rheumatol.

[9] Liu V, Fong W, Kwan YH, Leung YY (2021). Residual Disease Burden in patients with Axial Spondyloarthritis and Psoriatic Arthritis despite Low Disease Activity States in a multiethnic Asian Population. J Rheumatol.

[10] Ishida M, Kuroiwa Y, Yoshida E, Sato M, Krupa D, Henry N (2018). Residual symptoms and disease burden among patients with rheumatoid arthritis in remission or low disease activity: a systematic literature review. Mod Rheumatol.

[11] Nagy G, Roodenrijs NMT, Welsing PMJ, Kedves M, Hamar A, van der Goes MC (2022). EULAR points to consider for the management of difficult-to-treat rheumatoid arthritis. Ann Rheum Dis.

[12] Cook MJ, Diffin J, Scirè CA, Lunt M, MacGregor AJ, Symmons DPM (2016). Predictors and outcomes of sustained, intermittent or never achieving remission in patients with recent onset inflammatory polyarthritis: results from the Norfolk Arthritis Register. Rheumatol Oxf Engl.

[13] Lubrano E, Scriffignano S, Perrotta FM (2020). Residual Disease Activity and Associated Factors in psoriatic arthritis. J Rheumatol.

[14] Kerschbaumer A, Sepriano A, van der Smolen JS, Dougados M, van Vollenhoven R (2020). Efficacy of pharmacological treatment in rheumatoid arthritis: a systematic literature research informing the 2019 update of the EULAR recommendations for management of rheumatoid arthritis. Ann Rheum Dis.

[15] Sumpton D, Kelly A, Tunnicliffe DJ, Craig JC, Hassett G, Chessman D (2020). Patients’ perspectives and experience of Psoriasis and Psoriatic Arthritis: a systematic review and thematic synthesis of qualitative studies. Arthritis Care Res.

[16] Parenti G, Tomaino SCM, Cipolletta S (2020). The experience of living with rheumatoid arthritis: a qualitative metasynthesis. J Clin Nurs.

[17] Pearson NA, Tutton E, Martindale J, Strickland G, Thompson J, Packham JC (2022). Qualitative interview study exploring the patient experience of living with axial spondyloarthritis and fatigue: difficult, demanding and draining. BMJ Open.

[18] Bech B, Primdahl J, van Tubergen A, Voshaar M, Zangi HA, Barbosa L (2020). 2018 update of the EULAR recommendations for the role of the nurse in the management of chronic inflammatory arthritis. Ann Rheum Dis.

[19] Nikiphorou E, Santos EJF, Marques A, Böhm P, Bijlsma JW, Daien CI (2021). 2021 EULAR recommendations for the implementation of self-management strategies in patients with inflammatory arthritis. Ann Rheum Dis.

[20] Barlow J, Wright C, Sheasby J, Turner A, Hainsworth J (2002). Self-management approaches for people with chronic conditions: a review. Patient Educ Couns.

[21] Lorig KR, Holman H (2003). Self-management education: history, definition, outcomes, and mechanisms. Ann Behav Med Publ Soc Behav Med.

[22] Grønning K, Lim S, Bratås O (2020). Health status and self-management in patients with inflammatory arthritis—A five‐year follow‐up study after nurse‐led patient education. Nurs Open.

[23] Jensen NH, Aaby A, Ryom K, Maindal HT (2021). A CHAT about health literacy - a qualitative feasibility study of the Conversational Health Literacy Assessment Tool (CHAT) in a Danish municipal healthcare centre. Scand J Caring Sci.

[24] Urstad KH, Andersen MH, Larsen MH, Borge CR, Helseth S, Wahl AK (2022). Definitions and measurement of health literacy in health and medicine research: a systematic review. BMJ Open.

[25] Bandura A (1977). Self-efficacy: toward a unifying theory of behavioral change. Psychol Rev.

[26] Damgaard AJ, Primdahl J, Esbensen BA, Latocha KM, Bremander A (2023). Self-management support needs of patients with inflammatory arthritis and the content of self-management interventions: a scoping review. Semin Arthritis Rheum.

[27] Primdahl J, Latocha KM, Bremander A, Hendricks O, Østergaard M, Andersen L (2023). Development of an interdisciplinary nurse-coordinated self-management intervention (INSELMA) for patients with inflammatory arthritis.

[28] Skivington K, Matthews L, Simpson SA, Craig P, Baird J, Blazeby JM (2021). A new framework for developing and evaluating complex interventions: update of Medical Research Council guidance. BMJ.

[29] Hayes SC, Luoma JB, Bond FW, Masuda A, Lillis J (2006). Acceptance and commitment therapy: model, processes and outcomes. Behav Res Ther.

[30] Ibfelt EH, Jensen DV, Hetland ML (2016). The Danish nationwide clinical register for patients with rheumatoid arthritis: DANBIO. Clin Epidemiol.

[31] Maksymowych WP, Richardson R, van der Mallon C, Boonen A (2007). Evaluation and validation of the patient acceptable symptom state (PASS) in patients with ankylosing spondylitis. Arthritis Rheum.

[32] Joos E, Peretz A, Beguin S, Famaey JP (1991). Reliability and reproducibility of visual analogue scale and numeric rating scale for therapeutic evaluation of pain in rheumatic patients. J Rheumatol.

[33] Harris DR. Acceptance and Commitment Therapy (ACT). 2007;46.

[34] Elwyn G, Frosch D, Thomson R, Joseph-Williams N, Lloyd A, Kinnersley P (2012). Shared decision making: a model for clinical practice. J Gen Intern Med.

[35] Horn KK, Jennings S, Richardson G, van Vliet D, Hefford C, Abbott JH (2012). The patient-specific functional scale: Psychometrics, Clinimetrics, and application as a clinical outcome measure. J Orthop Sports Phys Ther.

[36] Klokkerud M, Dagfinrud H, Uhlig T, Dager TN, Furunes KA, Klokkeide Å (2018). Developing and testing a consensus-based core set of outcome measures for rehabilitation in musculoskeletal diseases. Scand J Rheumatol.

[37] de Wit MPT, Berlo SE, Aanerud GJ, Aletaha D, Bijlsma JW, Croucher L (2011). European League against Rheumatism recommendations for the inclusion of patient representatives in scientific projects. Ann Rheum Dis.

[38] Staniszewska S, Brett J, Simera I, Seers K, Mockford C, Goodlad S (2017). GRIPP2 reporting checklists: tools to improve reporting of patient and public involvement in research. Res Involv Engagem.

[39] Braun V, Clarke V (2006). Using thematic analysis in psychology. Qual Res Psychol.

[40] Braun V, Clarke V. Thematic analysis: a practical guide. SAGE; 2021. p. 377.

[41] Tong A, Sainsbury P, Craig J (2007). Consolidated criteria for reporting qualitative research (COREQ): a 32-item checklist for interviews and focus groups. Int J Qual Health Care.

[42] Grønning K, Rannestad T, Skomsvoll JF, Rygg LØ, Steinsbekk A (2014). Long-term effects of a nurse-led group and individual patient education programme for patients with chronic inflammatory polyarthritis - a randomised controlled trial. J Clin Nurs.

[43] Hewlett S, Almeida C, Ambler N, Blair PS, Choy E, Dures E (2019). Group cognitive-behavioural programme to reduce the impact of rheumatoid arthritis fatigue: the RAFT RCT with economic and qualitative evaluations. Health Technol Assess Winch Engl.

[44] Marques A, Santos E, Nikiphorou E, Bosworth A, Carmona L (2021). Effectiveness of self-management interventions in inflammatory arthritis: a systematic review informing the 2021 EULAR recommendations for the implementation of self-management strategies in patients with inflammatory arthritis. RMD Open.

[45] Latocha KM, Løppenthin KB, Østergaard M, Jennum PJ, Hetland ML, Røgind H (2023). The effect of group-based cognitive behavioural therapy for insomnia in patients with rheumatoid arthritis: a randomized controlled trial. Rheumatol Oxf Engl.

[46] Primholdt N, Primdahl J, Hendricks OA, Difficult Diagnosis (2017). A qualitative study of the Daily Lives of Young Men Diagnosed with Ankylosing Spondylitis. Musculoskelet Care.

[47] Feddersen H, Mechlenborg Kristiansen T, Tanggaard Andersen P, Hørslev-Petersen K, Primdahl J (2019). Juggling identities of rheumatoid arthritis, motherhood and paid work - a grounded theory study. Disabil Rehabil.

[48] Lorig KR, Sobel DS, Ritter PL, Laurent D, Hobbs M (2001). Effect of a self-management program on patients with chronic disease. Eff Clin Pract ECP.

[49] Primdahl J, Wagner L, Hørslev-Petersen K (2011). Being an outpatient with rheumatoid arthritis–a focus group study on patients’ self-efficacy and experiences from participation in a short course and one of three different outpatient settings. Scand J Caring Sci.

[50] Rees S, Williams A (2009). Promoting and supporting self-management for adults living in the community with physical chronic illness: a systematic review of the effectiveness and meaningfulness of the patient-practitioner encounter. JBI Libr Syst Rev.

[51] Fu Y, Yu G, McNichol E, Marczewski K, Closs SJ (2018). The association between patient-professional partnerships and self-management of chronic back pain: a mixed methods study. Eur J Pain Lond Engl.

[52] Coyne E, Carlini J, Doherty T, Harlow W, Mitchell ML, Grealish L (2020). Partnership between Nurse navigators and adult persons living with complex chronic disease-An exploratory study. J Clin Nurs.

[53] Smarr KL, Musser DR, Shigaki CL, Johnson R, Hanson KD, Siva C (2011). Online self-management in rheumatoid arthritis: a patient-centered model application. Telemed J E-Health off J Am Telemed Assoc.

[54] Chen SH, Yu KH, Lee YH, Shao JH (2022). Expectations of an online-self-management program for rheumatoid arthritis. Clin Nurs Res.

[55] Ayala GX, Elder JP (2011). Qualitative methods to ensure acceptability of behavioral and social interventions to the target population. J Public Health Dent.

[56] Block ES, Erskine L (2012). Interviewing by telephone: specific considerations, opportunities, and challenges. Int J Qual Methods.

[57] Malterud K, Siersma VD, Guassora AD (2016). Sample size in qualitative interview studies: guided by Information Power. Qual Health Res.

