# Supplementary material for: Patients’ experience of a novel interdisciplinary nurse-led self-management intervention (INSELMA)—a qualitative evaluation
Source: BMC Rheumatol. 2024 Mar 1;8:10. doi: 10.1186/s41927-024-00379-6 (PMC10905856; doi:10.1186/s41927-024-00379-6)
Supplement: Supplementary file 1 — Supplementary Material 1 [file 41927_2024_379_MOESM1_ESM.docx]

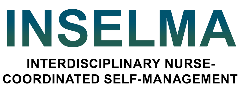
**INSELMA - Interview guide for individual interviews with patient participants after their last consultation**

**Aim:** To explore the patients’ experience of participating in the feasibility test of the INSELMA intervention

For interviewer: Following topics may guide the interviewer with main questions and examples of elaborate examples. Stay open to explore perspectives of importance to the participants.

INTRODUCTION

- **Presentation of interviewer**
- **Background and purpose of the interview**
- **Timeframe and clarification**. Max. 30-45 min. There are no wrong answers. Phones on silent mode
- **Confidentiality, anonymity, data security**

| BEFORE THE INSELMA INTERVENTION | | |
| --- | --- | --- |
| **What were your thoughts when entering the intervention?** | - What made you agree to participate in INSELMA? - What did you hope to get out of your participation before the intervention started? - What concerns did you have about your participation? How was it like to be presented with the project on the DANBIO screen? - How did you experience the written and oral information you received about the project? - Did you get an overall sense of what the project was about? |  |
| THE INITIAL CONSULTATION | | |
| **How did you experience the first consultation with the coordinating rheumatology nurse?** | - What was the focus of the interview? Did it align with what was important to you? - How did you experience the dialogue and the communication with the nurse? - (Did the nurse listen? Was there a focus on your needs and wishes?) - How did you agree on the plan and the process in terms of what you chose to work with in the study period? - How was it for you to focus on your wishes and to prepare specific goals in relation to activities? - What was it like to rate your activities? |  |
| DURING THE INTERVENTION PERIOD | | |
| **Did anything change for you during the intervention?**  **In that case, what do you consider made a difference for you?** | - How did you experience interacting/working with the same rheumatology nurse over a longer period? - What did the nurse focus on during the consultations? - What was Your focus during the period? What did You decide to work with? - How did you experience working with your goals / agreed activities both at home and at follow-up? - Was it meaningful for you to work that way? - What is your experience of your influence on the choices and decisions that were made along the way? - What significance did it have for you? - Has something changed for you during the project (e.g., need for support or what you would like to be able to do)? - Did you participate in the entire intervention? If not - what made you stop?   **Interdisciplinary and cross-sectoral:**   - How did you experience multi/interdisciplinary involvement? - Were there other health professionals you needed support from? - Have you been in contact with your general practitioner or other persons in the municipality as part of the project? - Did you experience a multidisciplinary meeting? **If yes:** Who attended (also relatives)? - How was it like to participate? What role and influence did you have? - How did you experience the communication in the meeting? |  |
| AFTER THE INTERVENTION | | |
| **What impact did the intervention have for you?** | - Did your participation in the INSELMA study make any difference to you? What difference can you feel? What do you think made a difference? - **If little or no significance from the intervention:**  Why do you think this is so? What would have made a difference? - Has anything changed in the way you think about your arthritis and your challenges? If so, how? - Is there anything in the way you manage your arthritis and symptoms now that is different compared to before participation in the intervention? Is there something you still think you need support or help with? - What do you think of the collaboration with the nurse / physiotherapist / occupational therapist? - Has the intervention changed anything regarding your everyday life, your family, your work, or your network? - How did the intervention differ from your "normal" outpatient consultations? Please share examples. |  |
| YOUR FUTURE |  |  |
| **How do you feel able to manage your IA and everyday life in the future compared to the time before participating in INSELMA?** | - How do you feel able to manage your arthritis and everyday life in the future compared to before your participation in the INSELMA study? - In what way were you prepared to what was to happen after the course ended? - How do you think you can move forward on your own after the project has finished? |  |
| OTHER CONSIDERATIONS | | |
| **What do you think could have been done differently?** | - Is there anything that surprised you about the process? - The duration was 6 months. What are your thoughts about that? - What do you think about the number of consultations you had? - What are your thoughts about the possibility to have consultations by phone/face to face /online? - What are your thoughts about receiving and completing the questionnaires via the link in your e-box? - Was there anything that surprised you by the questions? Anything missing? - Would you have liked to have an opportunity to meet with likeminded people with IA? (Group sessions) - What advice would you give us, if we were to adjust the intervention and offer it to more patients? - What was the best thing about being part of INSELMA? - What has been the most challenging being part of INSELMA? |  |
| FINAL REMARKS | - Do you have any questions? Is there anything you would like to elaborate on? - Thank you so much for coming and sharing your experiences |  |
